# On the Helix Propensity in Generalized Born Solvent Descriptions of Modeling the Dark Proteome

**DOI:** 10.3389/fmolb.2017.00003

**Published:** 2017-01-31

**Authors:** Mark A. Olson

**Affiliations:** Department of Cell Biology and Biochemistry, Molecular and Translational Sciences Division, United States Army Medical Research Institute for Infectious DiseasesFredrick, MD, USA

**Keywords:** molecular dynamics, free-energy landscape, intrinsically disordered proteins, explicit/implicit solvent model replica-exchange simulation

## Abstract

Intrinsically disordered proteins that populate the so-called “Dark Proteome” offer challenging benchmarks of atomistic simulation methods to accurately model conformational transitions on a multidimensional energy landscape. This work explores the application of parallel tempering with implicit solvent models as a computational framework to capture the conformational ensemble of an intrinsically disordered peptide derived from the Ebola virus protein VP35. A recent X-ray crystallographic study reported a protein-peptide interface where the VP35 peptide underwent a folding transition from a disordered form to a helix-β-turn-helix topological fold upon molecular association with the Ebola protein NP. An assessment is provided of the accuracy of two generalized Born solvent models (GBMV2 and GBSW2) using the CHARMM force field and applied with temperature-based replica exchange dynamics to calculate the disorder propensity of the peptide and its probability density of states in a continuum solvent. A further comparison is presented of applying an explicit/implicit solvent hybrid replica exchange simulation of the peptide to determine the effect of modeling water interactions at the all-atom resolution.

## Introduction

The large conformational heterogeneity and rapid dynamic transitions of intrinsically disordered peptides and proteins (IDPs) present a challenge to experimental boundaries in characterizing their functional form on rugged energy landscapes (Wright and Dyson, 1999, 2005). From a biological perspective, the broad interest in IDPs draws principally from their fundamental role in the regulation and function of cellular protein networks. Recent experimental studies have begun to unravel the interplay between “ordered chaos” of IDPs and their kinetic transition to a topological funnel of folded states (Arai et al., 2015). The contemporary view of this dynamic process is one that occurs by either an “induced-fit” of the IDP upon molecular association with a protein target or by target “fly casting” of a prefolded state in the disordered conformational ensemble of the IDP (see, e.g., Shoemaker et al., 2000; Arai et al., 2015).

Complementary to experimental studies are computer simulations which offer a powerful set of tools to understand IDPs at the all-atom level and their inherent plasticity to navigate a disordered network of microstates (see, e.g., Zhang and Chen, 2014; Chebaro et al., 2015; Bhowmick et al., 2016; Lee and Chen, 2016). Among the simulation methods, the generalized ensemble sampling technique of temperature-based replica exchange (T-ReX; Sugitaa and Okamoto, 1999; Ishikawa et al., 2001), also known as parallel tempering, has become an increasingly popular approach for exploring the energy landscape of proteins. Algorithms combined with T-ReX to generate protein configurations vary in their theoretical formulations and range from canonical molecular dynamics (MD) simulations to nontraditional methods that accelerate conformational sampling. Of the latter, examples includes coarse replica-exchange molecular dynamics (Peter et al., 2016), accelerated molecular dynamics (see, e.g., Miao et al., 2015), Hamiltonian switch Metropolis Monte Carlo (Mittal et al., 2014), all-atom multicanonical molecular dynamics (Higo et al., 2011) and self-guided Langevin dynamics (SGLD; Wu and Brooks, 2003), among others.

A computational strategy of reducing the complexity of all-atom simulations of proteins is the replacement of explicit water interactions with a continuum description of treating implicitly the bulk physical properties of solvation effects. The most common implicit solvent method for protein dynamics simulations is the generalized Born (GB) approximation. GB models are computationally faster than explicit solvent calculations and differ in their accuracy of reproducing Poisson-Boltzmann solvation energies for single protein conformations (see, e.g., Feig et al., 2004b). Application of GB solvent models to studies of IDPs has been reported by several laboratories (see, e.g., Ganguly and Chen, 2009; Click et al., 2010; Chebaro et al., 2015; Ganguly and Chen, 2015). To date the simulation results lack consensus on the accuracy of GB solvent models as a computational framework to capture the fold propensities of IDPs and their probability density of states on a conformational landscape. Particularly missing among the reported studies are comparative assessments of GB models of IDPs with those modeled by explicit all-atom solvent replica exchange simulations.

Given the current interests in characterizing the “Dark Proteome” which consists of “invisible” conformational states within the human, viral and microbial protein fold universe (Perdigão et al., 2015; Bhowmick et al., 2016), this work presents temperature-based replica exchange simulations of modeling an IDP derived from an Ebola virus protein. Ebola viruses are nonsegmented negative sense RNA viruses that cause severe hemorrhagic fever (Sanchez et al., 2006). An X-ray crystallographic structure was reported by Amarasinghe and coworkers (Leung et al., 2015) of the Ebola nucleoprotein NP in complex with a 28-residue peptide extracted from Ebola VP35 (peptide designated as NPBP). The NP-VP35 viral assembly is essential for virus replication and offers a protein target for therapeutic development. Experimental data reveals the NPBP peptide binds NP with high affinity and specificity, and acts by blocking NP oligomerization. The peptide undergoes a folding transition from a disordered form free in solution to a helix-β-turn-helix fold upon molecular association with NP (Leung et al., 2015).

Two different generalized ensemble sampling methods are applied based on combining T-ReX with the SGLD simulation method (Lee and Olson, 2010) and two different GB solvent models are examined to assess their accuracy in modeling the probability density of states of the NPBP peptide. One of the sampling methods is the conventional application of T-ReX with a static set of temperatures to explore the conformational landscape. The other technique is an adaptive T-ReX where the replica clients dynamically walk in temperature space in search of the optimal population density on a modeled energy function (Katzgraber et al., 2006; Trebst et al., 2006; Lee and Olson, 2011; Olson and Lee, 2014; Olson et al., 2016). The GB models analyzed are GBMV2 (generalized Born molecular volume; Lee et al., 2002, 2003) and the GBSW2 (generalized Born smoothing window; Im et al., 2003). The models differ in their dielectric-boundary descriptions with one of them constructed from an analytical formulation of the molecular volume (Lee et al., 2003).

The final simulation model applied to the NPBP peptide is an explicit/implicit solvent hybrid T-ReX/MD method (Chaudhury et al., 2012). The application of this simulation model is to investigate the effect of solvent resolution on the helix propensity and the search of conformational transitions. The idea behind the hybrid model is reducing the number of replica clients needed in explicit solvent simulations by replacing the contribution of explicit solvent energies in the Metropolis exchanges (Metropolis et al., 1953) with those of the GBMV2 solvent approximation. The hybrid model allows the same number of replica clients to be applied as in the GB solvent T-ReX/SGLD simulations of the NPBP peptide while retaining a higher resolution in conformational sampling on an explicit solvent landscape (Chaudhury et al., 2012; Olson and Lee, 2013).

## Computational methods

**Figure 1:**
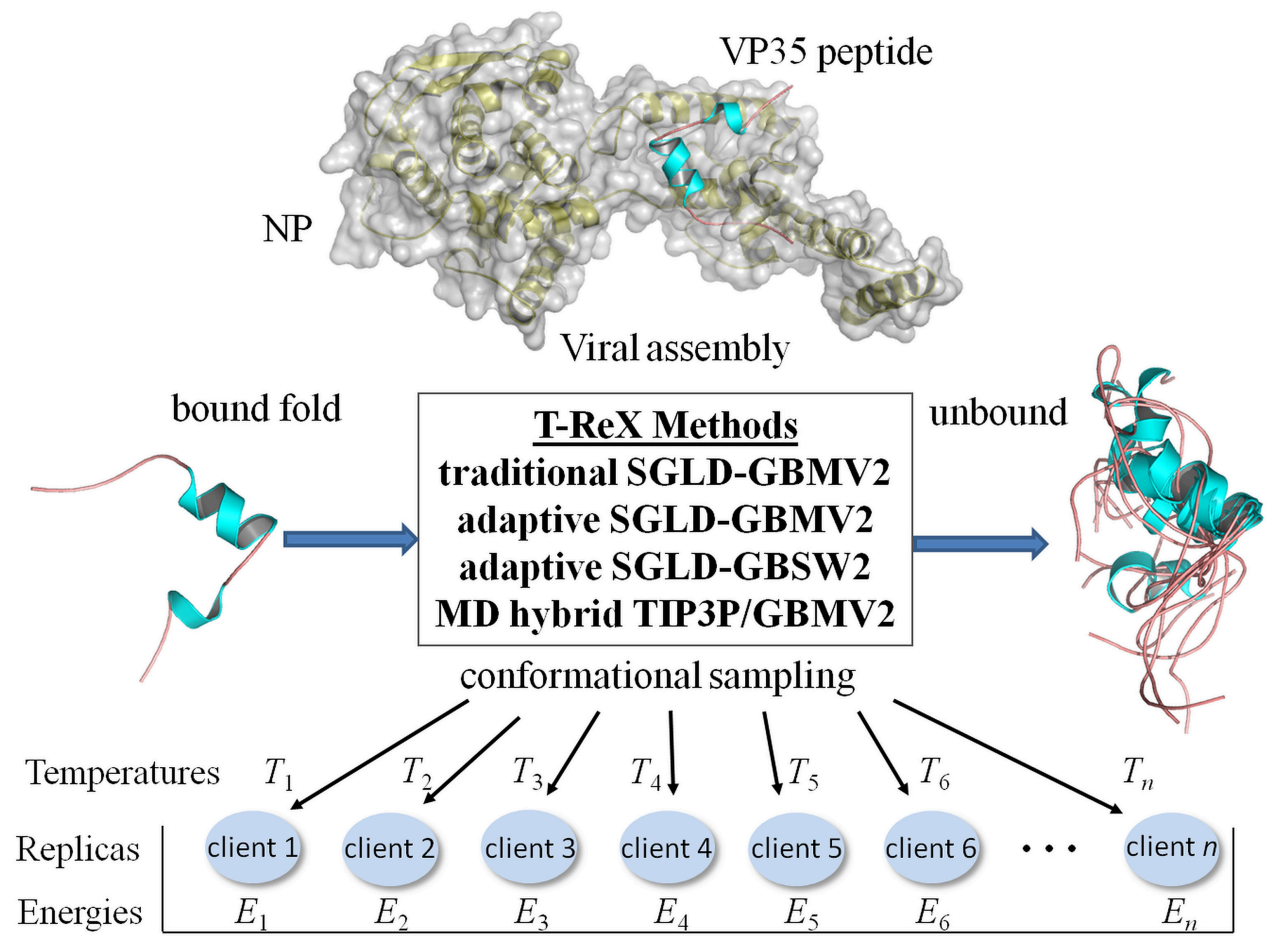
**Computational strategies of modeling the Ebola virus VP35 peptide (PDB: 4YPI) in its unbound form using temperature-based replica exchange (T-ReX) simulation methods**. The methods include: (1) GBMV2 solvent model applied with a traditional (static) set of temperatures spanning a range from a minimum temperature (*T*_1_) to the upper extreme (*T*_*n*_), where *n* is the number thermal windows (ensemble computing clients); (2) GBMV2 using an adaptive (dynamically walking) set of temperatures between *T*_1_and *T*_*n*_; (3) GBSW2 solvent model applied by adaptive sampling; and (4) TIP3P/GBMV2 hybrid replica exchange method. Energies (*E*_*i*_) used in the replica exchanges are described in the text. Molecular figures were drawn with PyMOL (www.pymol.org).

This section provides a brief outline of the computational methods applied in this work of modeling the NPBP peptide taken from the PDB 4YPI (Figure 1). Summarized are the sampling techniques and protocols as well as metrics to evaluate the simulation trajectories.

### Replica exchange schemes

A general approach for conformational sampling is the application of T-ReX (see, e.g., Ishikawa et al., 2001). Unlike the well-established method of MD simulations at a single sampling temperature, T-ReX is a generalized ensemble method of applying multiple parallel simulations in which each replica is executed at a different temperature. In traditional applications of T-ReX, the temperatures *T*_1_, *T*_2_, …, *T*_*n*_, where *n* is the number of replica clients, are predetermined by a static (fixed) set of values that span a desired range. It is common to model the set of temperatures by a geometrically spaced sequence (Predescu et al., 2004) using *n* − 1 intervals from the minimum temperature denoted as *T*_1_ = *T*_min_ to the maximum *T*_*n*_ = *T*_max_

(1) $$\begin{array}{r} T_{i\;+\;1}=T_{i}{\left(T_{\max}/T_{\min}\right)}^{\left[\frac{1}{n-1}\right]}, \end{array}$$

where *T*_*i*_ is the temperature of the *i*th replica client illustrated in Figure 1.

An alternative to Equation (1) is an adaptive replica exchange method of allowing the clients to dynamically walk in temperature space (Katzgraber et al., 2006; Trebst et al., 2006; Lee and Olson, 2011; Olson and Lee, 2014; Olson et al., 2016). In implementing the adaptive algorithm, each client is tagged as either “cold” or “hot” depending on the last temperature extreme it visited (Lee and Olson, 2011). Tracing of the clients is made by constructing histograms over temperature space, *n*_cold_(*T*) and *n*_hot_(*T*), where each bin accumulates the number of cold and hot clients visiting each temperature window. The fraction cold, *f*_cold_(*T*), of a client window at temperature *T* is the number of cold clients visiting that temperature divided by the total number of cold and hot client visits:

(2) $$\begin{array}{r} f_{\text{cold}}\left(T\right)=\frac{n_{\text{cold}}\left(T\right)}{n_{\text{cold}}\left(T\right)+n_{\text{hot}}\left(T\right)}. \end{array}$$

Using the *f*_cold_(*T*) term, a thermal current is defined (Lee and Olson, 2011)

(3) $$\begin{array}{r} j=D\left(T\right)\;\eta \;\left(T\right)\frac{df_{\text{cold}}\left(T\right)}{dT}, \end{array}$$

where *D*(*T*) is the diffusivity and η(*T*) is the probability that any client will reside at temperature *T*. The current *j* can be maximized by adjusting the temperatures such that *f*_cold_(*T*_*i*_) increases linearly as a function of temperature index, *i*. Here in this work, a continuous function is constructed from the computed values of *f*_cold_(*T*_*i*_) at the current set of temperatures, *T*_*i*_, and new temperatures are searched for where *f*_cold_(*T*_*i*_) = *i*/(*N*−1). To prevent all of the windows from clustering around the same temperature and depleting exchanges at the extremes, a constraint is applied where no neighboring temperatures can be more than two geometric spacing units apart,

(4) $$\begin{array}{r} \frac{T_{i\;+\;1}}{T}\leq {\left(\frac{T_{\max}}{T_{\min}}\right)}^{\left[\frac{2}{N-1}\right]} \end{array}$$

with the lower and upper values of *T*_*i*_ set to *T*_min_ and *T*_max_, respectively.

The exchange of temperatures between neighboring replica clients, *a* and *b*, is determined by the Metropolis energy criteria (Metropolis et al., 1953)

(5) $$\begin{array}{r} p\left(a↔b\right)=\min\left[1,e^{\left(\beta_{a}-\beta_{b}\right)\left(E_{b}-E_{a}\right)}\right], \end{array}$$

where β_a_ = 1/*k*_*B*_*T*_*a*_, *k*_*B*_ is Boltzmann's constant, *T*_*a*_ is the temperature of replica client *a*, and *E*_*a*_ is the potential energy of client *a*.

### SGLD simulation models

For generating trajectories of the NPBP peptide, two methods were combined with T-ReX. The first is based on the SGLD simulation method developed by Wu and Brooks (2003). The SGLD equation of motion is given by

(6) $$\begin{array}{r} {\dot{\text{p}}}_{\text{i}}={\text{f}}_{\text{i}}-\gamma_{\text{i}}{\text{p}}_{\text{i}}+{\text{R}}_{\text{i}}+\lambda {\text{g}}_{\text{i}}, \end{array}$$

where ${\dot{\text{p}}}_{i}$ defines the rate of change of the momentum of particle *i*, **f**_*i*_ is the force acting on the particle, γ_*i*_ is the friction constant, **R**_*i*_ defines the random force and **g**_*i*_ is a memory function, which is scaled by an *ad hoc* guiding factor λ. The memory function **g**_*i*_ is defined by the moving average of momentum over an interval of time, *L*:

(7) $$\begin{array}{r} {\text{g}}_{\text{i}}={\langle {\text{p}}_{\text{i}}\rangle }_{L}, \end{array}$$

where 〈…〉_*L*_ denotes a local average. The time interval is further defined as *L* = *t*_*L*_/δ*t*, where *t*_*L*_ is the local averaging time and δ*t* the time step along the simulation trajectory. It should be noted that because of the *ad hoc* force in Equation (6), the sampling algorithm deviates from a canonical ensemble (Lee and Olson, 2010; Wu and Brooks, 2011; Wu et al., 2012, 2016). For this work, the deviation is anticipated to be small for modeling a mini-protein (Lee and Olson, 2010), nevertheless the population distributions can be reweighted to remove the applied bias (Wu and Brooks, 2011).

In the SGLD simulations, solvent was represented by either the implicit solvent model GBMV2 (Lee et al., 2002, 2003) or GBSW2 (Im et al., 2003). The most noted difference between the two models is representation of the solvent excluded volume and the treatment of the dielectric interface. The GBMV2 parameters were selected to smooth the molecular volume by setting β_s_ = −12 and P3 = 0.65 (Yeh et al., 2008). The hydrophobic cavitation term was modeled by applying a phenomenological surface tension coefficient set to a value of 0.015 kcal/mol/Å^2^. For applying GBSW2, the model was parameterized to fit the Lee-Richards molecular-surface Poisson results and required *w* = 0.2 Å, *a*_0_ = 1.2045, and *a*_1_ = 0.1866. The hydrophobic cavitation-energy tension term was set to 0.030 kcal/(molÅ^2^).

The utilities and programming libraries of the Multiscale Modeling Tools for Structural Biology (MMTSB; Feig et al., 2004a) were used to carry out the T-ReX/SGLD simulations. The CHARMM simulation program (version c35b2) was applied as a modeling platform (Brooks et al., 2009). Simulations were carried out using 24 replica clients and the frequency of exchanges was set to every 1 ps of simulation. Temperatures were set at *T*_min_ = 300 K and *T*_max_ = 475 K. Because the implicit solvent models GBMV2 and GBSW2 were originally developed for and have been extensively benchmarked with the CHARMM22 force field, this force field was applied with the CMAP backbone dihedral cross-term extension (Mackerell et al., 2004). An integration time step of 2 fs was used and parameters for SGLD consisted of the friction constant set to γ of 1 ps^−1^ for all heavy atoms, the guiding factor λ to a value of 1, and the averaging time *t*_*L*_ was set to 1 ps. These values were taken from previous studies of the SGLD model (Lee and Olson, 2010, 2011; Olson and Lee, 2014). Non-bonded interaction cutoff parameters for electrostatics and vdW terms were set at a radius of 22 Å with a 2-Å potential switching function. Covalent bonds between the heavy atoms and hydrogen atoms were constrained by the SHAKE algorithm (Ryckaert et al., 1977). The NPBP peptide was modeled for 200 ns of simulation time per thermal window, generating an ensemble of 4.8 μs.

### Hybrid simulation model

The alternative method applied for generating trajectories of the NPBP peptide is an explicit/implicit solvent hybrid T-ReX/MD simulation (Chaudhury et al., 2012). In a typical explicit solvent T-ReX simulation the energies are given by

(8) $$\begin{array}{r} E_{\text{explicit}}=U_{\text{all-atom}}^{\text{prot}}+U_{\text{all-atom}}^{\text{prot}-\text{solv}}+U_{\text{all-atom}}^{\text{solv}-\text{solv}}, \end{array}$$

where the first term describes the protein potential energy for a CHARMM-based molecular mechanics force field, the second term is the explicit protein-solvent interactions followed by the explicit solvent-solvent interactions. The all-atom solvent-solvent energy term requires significant number of replica-exchange clients to achieve adequate Metropolis updates (Chaudhury et al., 2012). In the hybrid T-ReX method, the dynamics of each replica moves on an explicit solvent landscape. During a Metropolis update, all waters are removed from a replica and the solvent energy term of the replica is calculated using the grid-based GBMV2 solvent model

(9) $$\begin{array}{r} E_{\text{implicit}}=U_{\text{all-atom}}^{\text{prot}}+\Delta G_{\text{GBMV2}}^{\text{prot}-\text{solv}}, \end{array}$$

where $\Delta G_{\text{GBMV2}}^{\text{prot}-\text{solv}}$ is the free-energy term due to the implicit solvent contribution. After completion of the Metropolis exchanges, the explicit waters in each replica are replaced to their configurations prior to removal and the simulation continues according to Equation (8).

The NAMD code (Phillips et al., 2005) was applied for the 200-ns T-ReX/MD simulation with the CHARMM22+CMAP force field. The simulation cubic box size was set to 53.19 Å^3^ and the number of waters was 4796. For modeling the waters the TIP3P potential was applied (Jorgensen et al., 1983). Nose'-Hoover thermostat was applied with a temperature coupling constant of 50 kcal/s^2^. Given that the computational expense of the hybrid model relative to implicit solvent calculations is greater, the NAMD simulation parameters differ slightly from the T-ReX/SGLD simulations in that a smaller cutoff distance of 12 Å was applied with a switching distance of 8 Å. The integration time step remained identical to that used with the SGLD simulations and the SHAKE algorithm was similarly applied. Particle mesh Ewald was applied and combined with periodic boundary conditions.

### Evaluation metrics

To examine the trajectories generated by the simulations, the weighted histogram analysis method (WHAM; Ferrenberg and Swendsen, 1989; Kumar et al., 1992; Gallicchio et al., 2005) was applied to the data sets. The 2D density of states, Ω (q_1_, q_2_), for a molecular system, where q_1_ and q_2_ are a set of reaction coordinates of interest, is given by

(10) $$\begin{array}{r} \Omega \left({\text{q}}_{\text{1}},{\text{q}}_{\text{2}}\right)=\frac{\overset{R}{\underset{i\;=\;1}{\sum }}N_{i}\left({\text{q}}_{\text{1}},{\text{q}}_{\text{2}}\right)}{\overset{R}{\underset{j\;=\;1}{\sum }}n_{i}\;\exp\;\left(f_{i}-\beta_{i}E\right)}, \end{array}$$

where *n*_*j*_ is the number of data points in the *j*th simulation and β_*j*_ and *T*_*j*_ are Boltzmann's constant and temperature of the *j*th simulation, respectively. The function *N*_*i*_(q_1_, q_2_) is the histogram of (q_1_, q_2_) calculated from the *i*th simulation, and *f*_*j*_ is the scaled free energy obtained by solving the following equations self-consistently,

(11) $$\begin{array}{l} P_{\beta }\left({\text{q}}_{\text{1}},{\text{q}}_{\text{2}}\right)=\frac{\overset{R}{\underset{i\;=\;1}{\sum }}N_{i}\left({\text{q}}_{\text{1}},{\text{q}}_{\text{2}}\right)\;\exp\;\left(-\beta E\right)}{\overset{R}{\underset{j\;=\;1}{\sum }}n_{i}\;\exp\;\left(f_{i}-\beta_{i}E\right)} \end{array}$$

and

(12) $$\begin{array}{l} \exp\;\left(-f_{i}\right)=\underset{{\text{q}}_{\text{1}},{\text{q}}_{\text{2}}}{\sum }\Omega \left({\text{q}}_{\text{1}},{\text{q}}_{\text{2}}\right)\;\exp\;\left(-\beta E\right), \end{array}$$

where *P*_β_(q_1_, q_2_) is the probability density at the inverse temperature β. From a density profile, a potential of mean force is determined from the relationship *W*_*T*_(q_1_, q_2_) = −R*T*log*P*_β_(q_1_, q_2_), where R is the universal gas constant. For calculations presented here, q_1_ = fractional helicity (*f*_H_) of the peptide determined from DSSP (Kabsch and Sander, 1983) and q_2_ = radius of gyration (*R*_g_).

The trajectories were further analyzed by a *Q* score for the peptide. *Q* is the number of side-chain contacts in a generated conformation divided by the total number equivalent contacts in the X-ray crystal structure of NPBP. Values were computed for side-chain center-of-mass pairs (*i*,*j*), such that *j* > *i* and whose distances are less than a cutoff of 4.2 Å. A sigmoidal function was applied (implemented in MMTSB) to effectively include residue pairs that are slightly further apart with a reduced weight. In addition to a *Q* score, pairwise Cα root-mean-square-deviation (RMSD) from the starting X-ray structure was computed for each peptide conformation in a generated ensemble of structures.

## Results and discussion

### Bound and free NPBP

**Figure 2:**
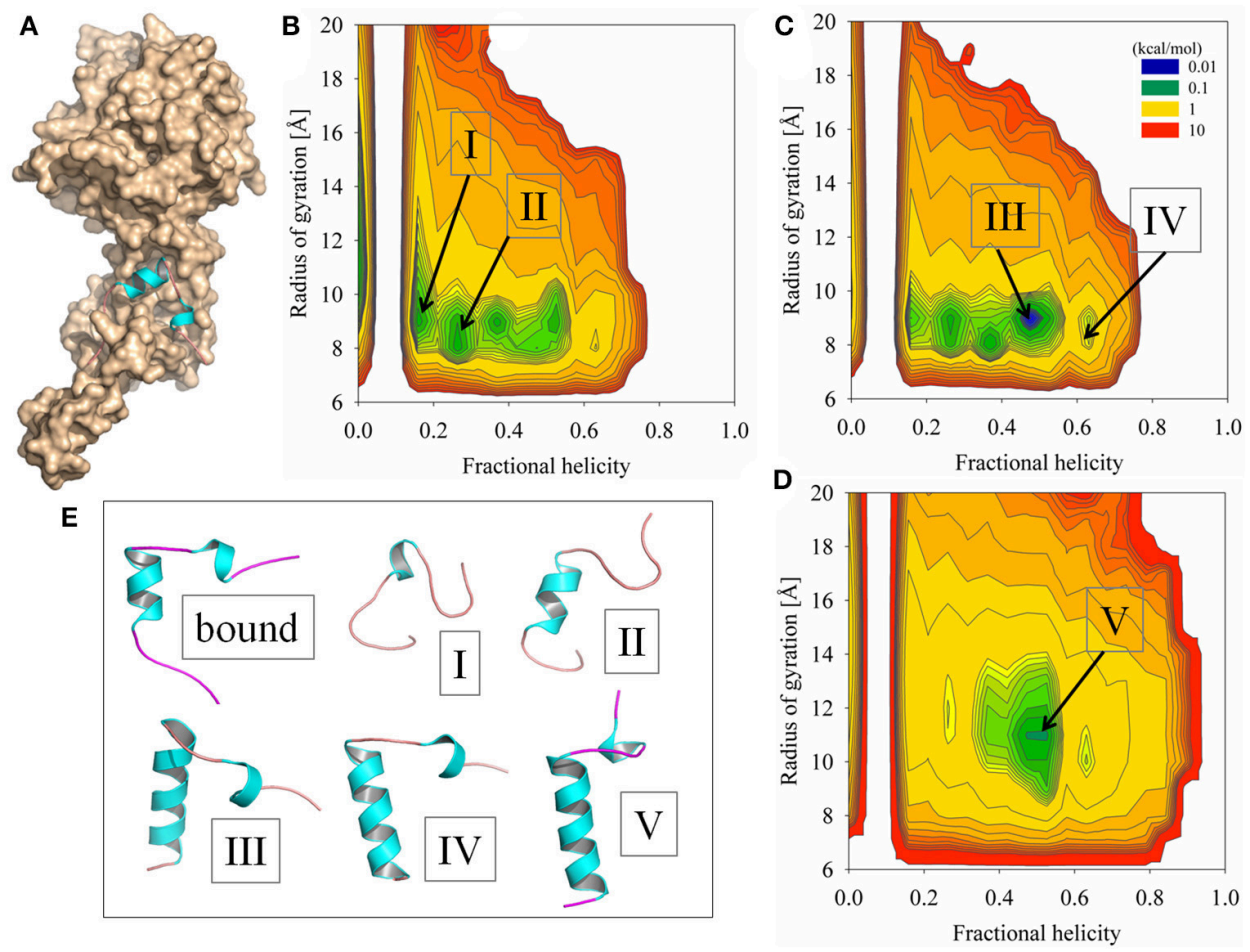
**Simulation results of sampling the Ebola virus VP35 NPBP peptide using GB-based solvent models combined with replica exchange methods. (A)** X-ray crystallographic structure of the NPBP peptide bound to the Ebola NP (displayed as a molecular surface). **(B)** Probability density profile *W*_*T*_(*f*_H_, R_g_) computed at *T* = 300 K and taken from the conformational ensemble modeled by the GBMV2 static T-ReX simulation method. The order parameters are fractional helicity and radius of gyration. **(C)** Probability density profile at *T* = 300 K from the adaptive T-ReX method with the GBMV2 solvent model. **(D)** Adaptive T-ReX with GBSW2 solvent model at the identical temperature. **(E)** Representative conformations extracted at *T* = 300 K from the simulations and are annotated at the indicated basins.

Figure 2 illustrates the X-ray crystallographic structure of the NPBP peptide extracted from the Ebola virus VP35 in association with the Ebola NP protein (Leung et al., 2015). The binding of NPBP occupies a functionally critical site on NP required for RNA synthesis. The peptide conformation is stabilized by a network of electrostatic interactions dominated by NP residues Arg240, Lys248, and Asp252. Using the DSSP secondary structure algorithm, NPBP (annotated as residues 20–47) shows segments Trp28 to Thr35 and Val40 to Asp42 as distinct helical conformations. The overall *f*_H_ is 0.4 and the bound form exhibits an *R*_g_ of 10.5 Å.

Experimental characterization of the secondary structure of the NPBP peptide free in solution by circular dichroism (CD) spectroscopy is reported to show the peptide as intrinsically disordered (Leung et al., 2015). When added to a solution of 50% trifluoroethanol (TFE), the NPBP peptide transitions from a coil to helical structures of ~30–40% helicity, thus suggesting a strong underlying secondary-structure propensity. Predictions of secondary-structure without bias of the crystallographic structure estimate the NPBP peptide to encompass a consensus *f*_H_ ~0.3 with probabilities >0.9 for helical formation in the sequence segment of Gly27 to Met34 (see, e.g., Kieslich et al., 2016).

### Implicit solvent T-ReX simulations

To examine the accuracy of implicit solvent models to counterbalance the network of electrostatic interactions of the viral assembly interface that contribute to the stabilization of the NPBP helical fold and produce a conformational landscape with a predisposed helix propensity in bulk water, replica-exchange simulations were performed using different simulation strategies. The conformational sampling approach of SGLD was explored with two different GB solvent models and two different temperature-based replica-exchange methods. The first simulation model result shown in Figure 2B is the SGLD-GBMV2 with a static (fixed) set of temperatures in defining the replica-exchange protocol. The 2D profile *W*_*T*_(*f*_H_, R_g_) computed at *T* = 300 K using WHAM of the full ensemble shows a large manifold of conformational substates with a helix distribution of *f*_H_ ~0–0.5. Several representative structures extracted from the basins are illustrated in Figure 2E. The conformational density takes place in *R*_g_ space of ~8–11 Å and at the lower end of the population distribution non-structured states are observed to occupy a large range of *R*_g_ values and show the canonical feature of disorder.

Given the broad population distribution produced by a static set of temperatures in the T-ReX simulations, it is important to test whether the simulation model provided optimal sampling of the basins. To address this issue, an adaptive replica-exchange SGLD-GBMV2 simulation model was applied whereby allowing the clients to walk in temperature space to optimize the efficiency of exchanges between nearest-neighbor thermal windows at potential energy barriers separating conformational basins (Lee and Olson, 2011; Olson and Lee, 2014; Olson et al., 2016). The 2D profile from the adaptive T-ReX is illustrated in Figure 2C for *T* = 300 K and the result is shown to retain the manifold of transient states similar to those sampled by the static T-ReX method, yet a population shift is observed toward an *f*_H_ ~0.5 at the cost of reducing the density of unstructured conformations. The theoretical goal of the adaptive method is to enhance sampling of conformational transitions for a modeled potential energy surface. Early success of the method applied to a sharp phase transition of unfolding-folding of the protein SH3 showed better agreement with the experimental melting temperature than the traditional static approach (Lee and Olson, 2011). In addition, the adaptive method captured with greater accuracy the native state of SH3 extracted from the conformational ensemble. Given these earlier outcomes, and while the NPBP certainly lacks the folding cooperativity of SH3, the result suggests for the CHARMM22+CMAP/GBMV2 potential energy surface a NPBP “native” state of helix propensity near the value observed experimentally for the crystallographic bound conformation. Although the simulation shows a high rate of transitions among different basins, the overall population weight is inconsistent with the CD analysis in free solution. Because the potential energy surface is identical between the static and adaptive T-ReX methods, the less-efficient sampling approach will eventually converge to find a comparable *W*_*T*_(*f*_H_, R_g_).

To determine the bias of the GBMV2 solvent approximation on *W*_*T*_(*f*_H_, R_g_), adaptive T-ReX simulations were performed with a different implicit solvent model based on the GBSW2 approximation. Of the GB-based solvent models developed for protein dynamics, GBMV2 is one of the most accurate models in reproducing Poisson-Boltzmann theory with a Lee-Richards molecular surface (Feig et al., 2004b). The basis of GBMV2 is an analytical formulation of the molecular volume (Lee et al., 2003), while the less accurate but computationally much faster GBSW2 model is based on a smooth dielectric-boundary formulation constructed by applying a superposition of atomic-centered polynomials (Im et al., 2003). The dissimilarities between the two models in conformational sampling are clearly illustrated in Figure 2D. Application of GBSW2 significantly reduces the number of high-probability conformational excursions and leads to a folding funnel at *f*_H_ ~0.5. While the “optimized” *f*_H_ from the two different implicit solvent models is surprisingly similar, the limited disorder from the GBSW2 model in its current parameterization makes this solvent approximation less suitable for modeling IDPs (for an alternative parameterization of GBSW, see, e.g., Chen, 2010).

**Figure 3:**
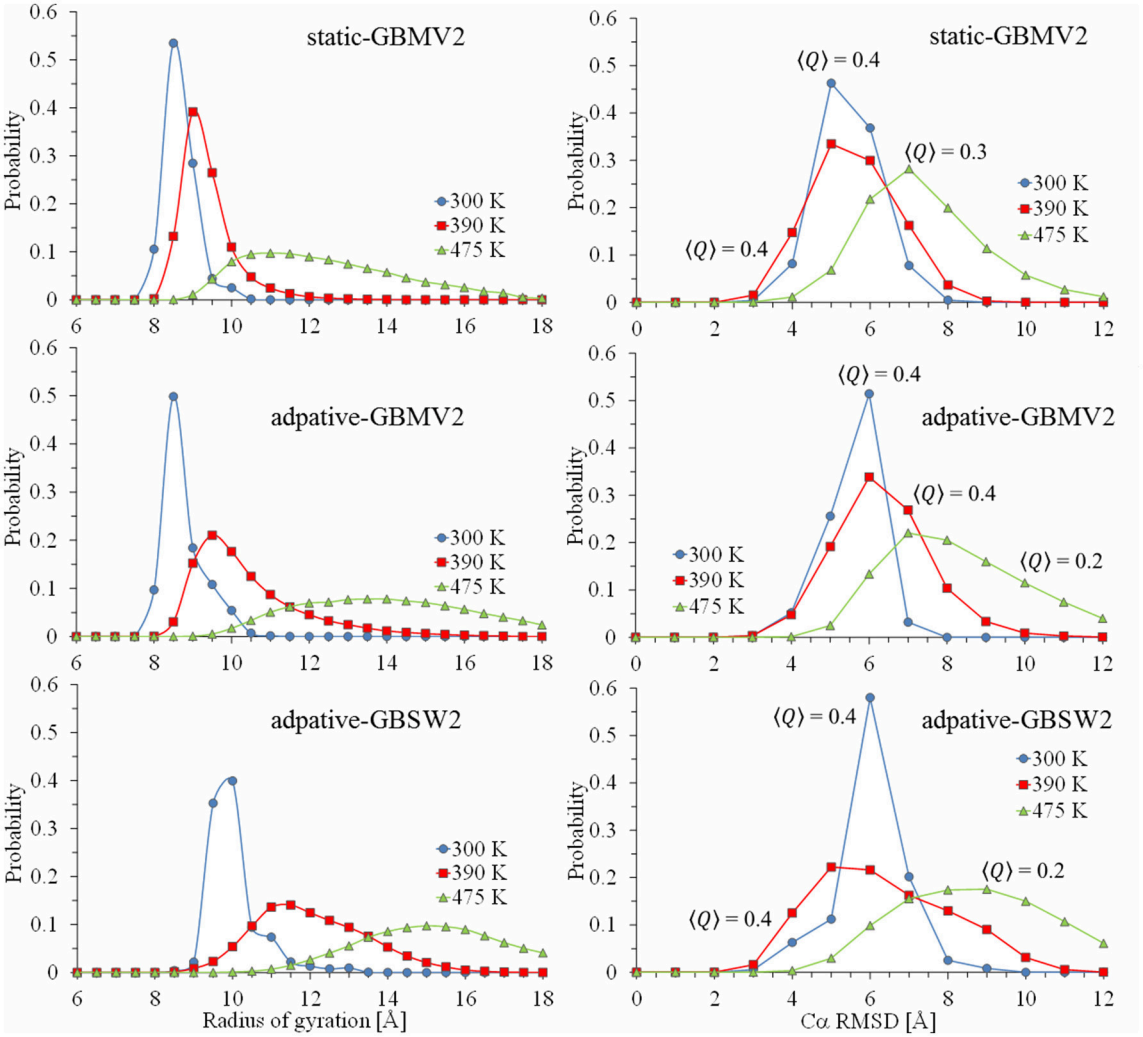
**Calculated probability profiles for sampling values of radius of gyration and Cα-RMSD from the starting bound conformation of the NPBP peptide**. Plot lines colored blue represent quantities extracted at *T* = 300 K from the generated conformational ensembles, red represent values at 390 K and green at 475 K. From the top figure to bottom, simulation results are static T-ReX/GBMV2, adaptive T-ReX/GBMV2, and adaptive T-ReX/GBSW2.

Figure 3 shows the probabilities of observing *R*_g_ as a function of three sampling temperatures taken from the ensemble. The GBMV2 model produced more compact states of NPBP than the crystallographic bound form, while GBSW2 yielded *R*_g_ values near the bound conformation. The observed difference between the solvent models can be partly attributed to the distinction in molecular surface representations, where different weights are applied to the surface-tension term that describes the hydrophobic free energy. In general, MD simulations of unfolded states are more compact and tend to favor helical structures than those found experimentally (Piana et al., 2014). By example, an experimental *R*_g_ for a unfolded 28 amino acids is estimated to be 13 Å (Kohn et al., 2004).

Also shown in Figure 3 are the probability profiles of C_α_-RMSD and the fraction of side-chain contacts similar to the starting conformation of NPBP. The ensemble average over contacts is denoted as <*Q*> and values <0.6 are considered unrelated to the starting structure. When combined with the analysis of the 2D profiles, the probabilities provide an interesting picture of the rare event of recognizing (via fly casting) a peptide conformation in the ensemble that is similar to the NPBP bound form. For the GBMV2 model and considering only the last 50 ns of simulation time, the lowest RMSD is 2.9 Å with *Q* = 0.6, and is clustered in the outer periphery of the highly-populated basin labeled as III in Figure 2C. This sparse cluster of low-RMSD states emerges with an *f*_H_ of 0.5 and *R*_g_ approaching 10 Å.

**Figure 4:**
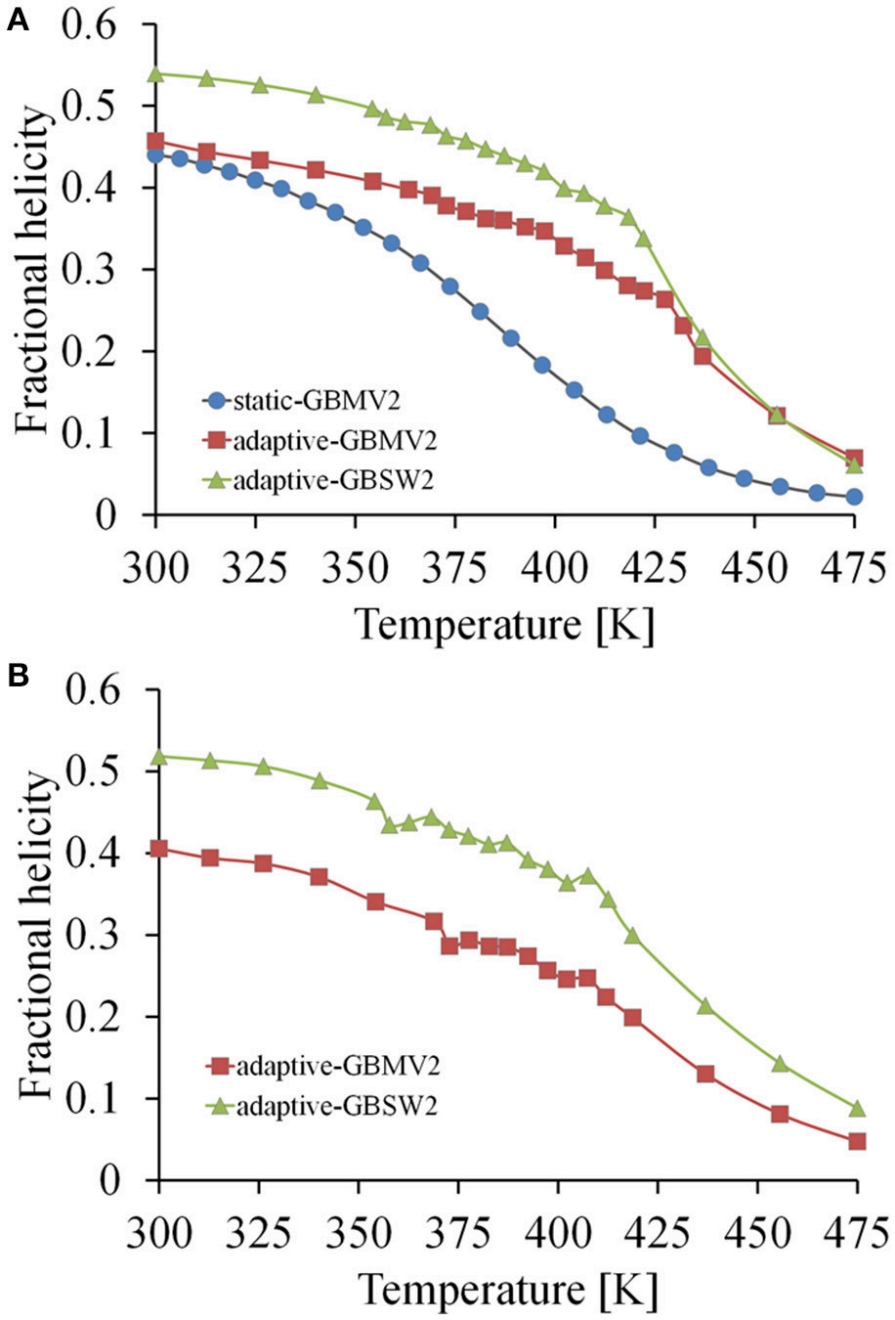
**Thermal unfolding profiles computed from the simulations of the Ebola VP35 NPBP peptide. (A)** Profiles calculated from the starting folded conformation using the three simulation models of the static T-ReX/GBMV2 (blue colored line), adaptive T-ReX/GBMV2 (red colored line), and adaptive T-ReX/GBSW2 (green colored line). **(B)** Profiles calculated from the adaptive T-ReX simulations of starting from an unstructured (coil) peptide fold.

It is also important to understand the configurational stability of IDPs from the simulations and their fold propensities. The thermal unfolding profiles for NPBP are shown in Figure 4A. Consistent with the reduced number of transient states and their populations among the GB models, GBSW2 retains helicity over a greater thermal range. The aggregation of replica clients in the range of 360 K–425 K for the adaptive method (GBMV2 and GBSW2) is the effect of enhanced sampling of unfolding-folding transition points that stabilize helix formation. The statistical errors in the histograms for all model simulations are approximately *f*_H_ ±0.1 along the temperature profiles. Simulation convergence and the dominance of helix formation in NPBP can be further tested by conducting T-ReX simulations starting from a random coil state rather than the folded conformation. Although these additional simulations were executed only to 100 ns using the adaptive method, Figure 4B shows convergence to a folded state of helical conformations and establishes the strong helix propensity of applying CHARMM22+CMAP/GB descriptions.

### Explicit/implicit solvent hybrid T-ReX/MD simulation

The overweighting of secondary structure biases from the GBMV2 and GBSW2 solvent models is comparable to other studies of using different GB solvent models and parameterizations (Ganguly and Chen, 2009; Click et al., 2010; Chebaro et al., 2015). As a further test of the impact of the GBMV2 solvent model and its mean-field resolution of smearing out the details of the solvent on sampling conformational transitions of NPBP, the final simulation model tested is the explicit/implicit solvent hybrid T-ReX/MD method. This model generates peptide configurations on an explicit solvent (TIP3P) landscape while using the same number of replica clients as in the implicit solvent calculations. The latter is achieved by using the GBMV2 model in the Metropolis exchanges rather than explicit solvent. While the goal is to evaluate the simulation model in terms of a conformational landscape rather than unconstrained folding free energies to high accuracy, it is worth noting that replacement of energies in the Metropolis updates from an all-atom representation to a mean-field approximation can produce errors in the detailed balance required of a canonical ensemble (Chaudhury et al., 2012).

**Figure 5:**
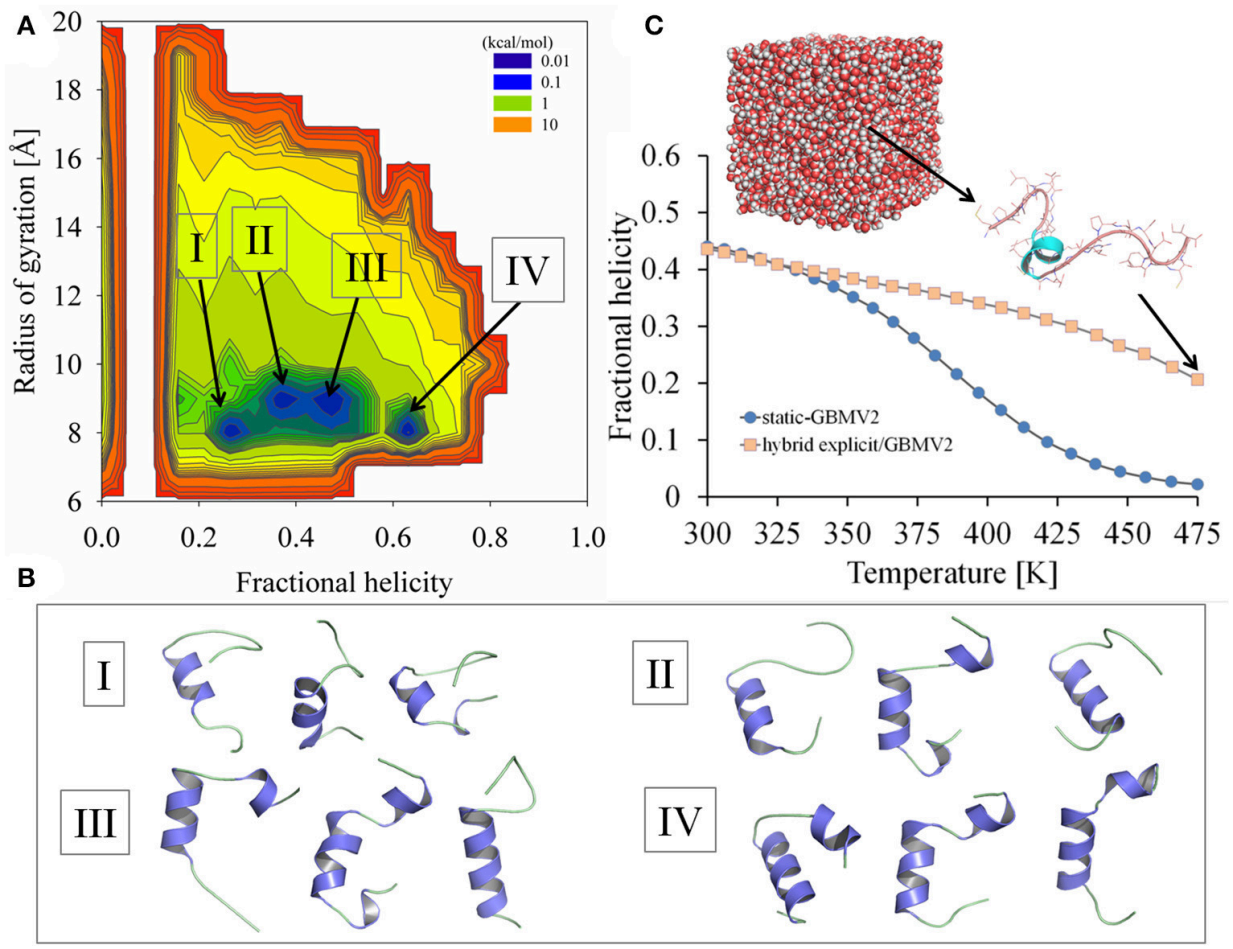
**Simulation results of sampling the Ebola virus VP35 NPBP peptide using the explicit/implicit solvent hybrid T-ReX/MD method. (A)** Probability density profile *W*_*T*_(*f*_H_, R_g_) computed at *T* = 300 K from sampling fractional helicity and radius of gyration. **(B)** Representative conformations extracted from the simulations are illustrated for selected basins. **(C)** Thermal unfolding profiles of the peptide computed using the explicit/implicit solvent hybrid T-ReX/MD method (light colored symbols) compared to the static T-ReX/SGLD method using GBMV2 (blue colored symbols). A representative structure is shown from the explicit solvent calculation.

Figure 5 shows *W*_*T*_(*f*_H_, R_g_) at *T* = 300 K from the WHAM calculation of the hybrid simulation model ensemble and the thermal unfolding profile. Several important observations can be made in comparison to the static GBMV2 model which best corresponds to the non-adaptive hybrid model. The most important distinction between the results is the striking difference in the favorable free energies and the network that shuttles conformations among the helical basins. While both sampling methods show sufficient plasticity among the states, the hybrid model shows a more quantifiable free-energy minimum at *f*_H_ = 0.26 vs. 0.37 for the static GBMV2, and yields good agreement with secondary-structure predictions. The distinction in the potentials of mean force among the models is illustrated by considering a transition between an unstructured state and the free-energy minimum. For the static GBMV2, the transition (*f*_H_ = 0; *R*_g_ = 11 Å) → (*f*_H_ = 0.37; *R*_g_ = 8 Å) yields ΔG = −0.1 kcal/mol, whereas for the adaptive model the transition from the same disordered state → (*f*_H_ = 0.47; *R*_g_ = 9 Å) ΔG = −1.0 kcal/mol, and for the hybrid model the transition → (*f*_H_ = 0.26; *R*_g_ = 9 Å) yields ΔG = −1.7 kcal/mol. Even though the static model exhibits a low-energy reversible transition to unstructured states and would appear to be in better agreement with the CD experiments (Leung et al., 2015), enhanced sampling of *P*_β_(*f*_H_, R_g_) by the adaptive method for this solvent description revealed a more costly transition to the densely populated *f*_H_ ~ 0.5.

The lowest RMSD conformer for the hybrid model via the last 50 ns is 3.3 Å with *Q* = 0.6 and *R*_g_ = 9.4 Å. This conformer is illustrated in Figure 5B as the first structure depicted for the basin labeled III. The conformation is formed from a helical hairpin of residues Ser26-Met34 and Val40-Phe44. The top-rank conformer based on potential energies for the free-energy minimum at *f*_H_ = 0.26 is illustrated as the first structure for basin I. This structure shows a 5-residue helix of Trp28-Met34. Among the highly populated basins, a distinction between the simulation models is the cluster at *f*_H_ = ~0.6, where the hybrid model shows an improved free energy of population. Unlike the other basins, this basin lacks a direct low-energy pathway along the manifold of clusters.

A statistical average of the ensemble for the hybrid model computed from the multiple temperatures of the T-ReX simulation is illustrated in Figure 5C along with a comparison with the static GBMV2 model. Despite the differences in the potentials of mean force between the models, a simple statistical average without reweighting based on free energies shows remarkably similar *f*_H_ values at 300 K. Because of the lack of instantaneous relaxation of the explicit waters in contrast to GB approximations, the hybrid model shows a reduction in excursions of unfolded states at the upper *R*_g_ boundaries. Like many MD simulations of unfolded states with explicit solvent (Piana et al., 2014), a residual secondary-structure propensity is observed at 475 K.

The more compact favorable states observed in the explicit/implicit solvent hybrid model than that corresponding to the bound NPBP conformation is unlikely due entirely to the GB model, but rather the additive force field (Piana et al., 2014). As noted above, the CHARMM22+CMAP force field was selected because of extensive benchmarks in reported studies of the GBMV2 and GBSW2 solvent descriptions to successfully model natively folded structures of proteins (see e.g., Yeh et al., 2008; Lee and Olson, 2010). While there are no reported studies of applying either GBMV2 or GBSW2 with the more refined CHARMM36m force field and its parameterization for TIP4P-based explicit solvent simulations (Huang et al., 2017), switching to this description may help reconcile the underestimated *R*_g_ values with those experimentally determined for unfolded states and reduce the overall weight and stabilization of secondary-structure propensities.

## Conclusions

The current initiative to develop an atomistic understanding of “invisible” conformational states of the human/viral/bacterial proteomes requires an accurate computational framework for modeling conformational transitions within a disordered ensemble and their population density. The work presented here examined the application of temperature-based replica exchange simulations with different sampling methods and solvent descriptions of modeling an intrinsically disorder 28-residue peptide from the Ebola virus protein VP35. The X-ray crystallographic determination of the VP35 peptide bound to Ebola NP reports a helix-β-turn-helix fold of roughly 40% helical structure, whereas from CD experiments in free solution the peptide is unstructured. The simulations of the unbound peptide showed the selection of a GB solvent model combined with a replica-exchange sampling protocol can have a significant effect on the distribution of sampled populations. Overall, the tested GB models tend to favor a free-energy minimum of roughly 50% helical content for the peptide. The effect of an adaptive temperature-based replica exchange protocol compared to a traditional approach of a static set of temperatures was found to reduce the amount of unstructured states and shifted the ensemble to helical conformations with an extended peptide folding stabilization. A comparison with an explicit/implicit solvent hybrid MD-based replica exchange simulation showed that conformational sampling on an explicit solvent landscape leads to a free-energy minimum of ~20% helicity, yet the overall conformational network underlying transient states resembles more of a helix-fold propensity in a solvent mixture of TFE-water rather than bulk water. The simulation results can be summarized as a benchmark for the testing of more refined CHARMM-based force fields and different GB model parameterizations. The ultimate goal is to capture greater heterogeneity in conformational probabilities and reduce the over-stabilization of helix propensities in modeling intrinsically disordered peptides.

## Author contributions

The author confirms being the sole contributor of this work and approved it for publication.

## Funding

Financial support for this work comes from US Department of Defense Threat Reduction Agency grant (DTRA 4.10011_07_RD_B). The opinions or assertions contained herein are the private views of the author and are not to be construed as official or as reflecting the views of the US Army or of the US Department of Defense. This article has been approved for public release with unlimited distribution.

### Conflict of interest statement

The author declares that the research was conducted in the absence of any commercial or financial relationships that could be construed as a potential conflict of interest.

## References

[B1] Arai M. Sugase K. Dyson H. J. Wright P. E. (2015). Conformational propensities of intrinsically disordered proteins influence the mechanism of binding and folding. Proc. Natl. Acad. Sci. U.S.A. 112, 9614–9619. 10.1073/pnas.1512799112 26195786 PMC4534220

[B2] Bhowmick A. Brookes D. H. Yost S. R. Dyson H. J. Forman-Kay J. D. Gunter D.. (2016). Finding our way in the dark proteome. J. Am. Chem. Soc. 138, 9730–9742. 10.1021/jacs.6b06543 27387657 PMC5051545

[B3] Brooks B. R. Brooks C. L. III. Mackerell A. D. Jr. Nilsson L. Petrella R. J. Roux B.. (2009). CHARMM: the biomolecular simulation program. J. Comput. Chem. 30, 1545–1614. 10.1002/jcc.21287 19444816 PMC2810661

[B4] Chaudhury S. Olson M. A. Tawa G. Wallqvist A. Lee M. S. (2012). Efficient conformational sampling in explicit solvent using a hybrid replica exchange molecular dynamics method. J. Chem. Theory Comput. 8, 677–687. 10.1021/ct200529b 26596615

[B5] Chebaro Y. Ballard A. J. Chakraborty D. Wales D. J. (2015). Intrinsically disordered energy landscapes. Sci. Rep. 5:10386. 10.1038/srep10386 25999294 PMC4441119

[B6] Chen J. (2010). Effective approximation of molecular volume using atom-centered dielectric functions in generalized Born models. J. Chem. Theory Comput. 6, 2790–2803. 10.1021/ct100251y 26616080

[B7] Click T. H. Ganguly D. Chen J. (2010). Intrinsically disordered proteins in a physics-based world. Int. J. Mol. Sci. 11, 5292–5309. 10.3390/ijms11125292 21614208 PMC3100817

[B8] Feig M. Karanicolas J. Brooks C. L. III. (2004a). MMTSB Tool Set: Enhanced sampling and multiscale modeling methods for applications in structural biology. J. Mol. Graph. Model. 22, 377–395. 10.1016/j.jmgm.2003.12.005 15099834

[B9] Feig M. Onufriev A. Lee M. S. Im W. Case D. A. Brooks C. L. III. (2004b). Performance comparison of generalized born and poisson methods in the calculation of electrostatic solvation energies for protein structures. J. Comput. Chem. 25, 265–284. 10.1002/jcc.10378 14648625

[B10] Ferrenberg A. M. Swendsen R. H. (1989). Optimized Monte Carlo data analysis. Phys. Rev. Lett. 63, 1195–1198. 10.1103/PhysRevLett.63.1195 10040500

[B11] Gallicchio E. Andrec M. Felts A. K. Levy R. M. (2005). Temperature weighted histogram analysis method, replica exchange, and transition paths. J. Phys. Chem. B. 109, 6722–6731. 10.1021/jp045294f 16851756

[B12] Ganguly D. Chen J. (2009). Atomistic details of the disordered states of KID and pKID. Implications in coupled binding and folding. J. Am. Chem. Soc. 131, 5214–5223. 10.1021/ja808999m 19278259

[B13] Ganguly D. Chen J. (2015). Modulation of the disordered conformational ensembles of the p53 transactivation domain by cancer-associated mutations. PLoS Comput. Biol. 11:e1004247. 10.1371/journal.pcbi.1004247 25897952 PMC4405366

[B14] Higo J. Nishimura Y. Nakamura H. (2011). A free-energy landscape for coupled folding and binding of an intrinsically disordered protein in explicit solvent from detailed all-atom computations. J. Am. Chem. Soc. 133, 10448–10458. 10.1021/ja110338e 21627111

[B15] Huang J. Rauscher S. Nawrocki G. Ran T. Feig M. de Groot B. L.. (2017). CHARMM36m: an improved force field for folded and intrinsically disordered proteins. Nat. Methods 14, 71–73. 10.1038/nmeth.4067 27819658 PMC5199616

[B16] Im W. Lee M. S. Brooks C. L. III. (2003). Generalized born model with a simple smoothing function. J. Comput. Chem. 24, 1691–1702. 10.1002/jcc.10321 12964188

[B17] Ishikawa Y. Sugita Y. Nishikawa T. Okamoto Y. (2001). Ab initio replica-exchange monte carlo method for cluster studies. Chem. Phys. Lett. 33, 199–206. 10.1016/S0009-2614(00)01342-7

[B18] Jorgensen W. L. Chandrasekhar J. Madura J. D. Impey R. W. Klein M. L. (1983). Comparison of simple potential functions for simulating liquid water. J. Chem. Phys. 79, 926–935. 10.1063/1.445869

[B19] Kabsch W. Sander C. (1983). Dictionary of protein secondary structure: pattern recognition of hydrogen-bonded and geometrical features. Biopolymers 22, 2577–2637. 10.1002/bip.360221211 6667333

[B20] Katzgraber H. G. Trebst S. Huse D. A. Troyer M. (2006). Feedback-optimized parallel tempering Monte Carlo. J. Stat. Mech. Theory Exp. 2006:P03018 10.1088/1742-5468/2006/03/p03018

[B21] Kieslich C. A. Smadbeck J. Khoury G. A. Floudas C. A. (2016). conSSert: consensus SVM model for accurate prediction of ordered secondary structure. J. Chem. Inf. Model. 56, 455–461. 10.1021/acs.jcim.5b00566 26928531

[B22] Kohn J. E. Millett I. S. Jacob J. Zagrovic B. Dillon T. M. Cingel N.. (2004). Random-coil behavior and the dimensions of chemically unfolded proteins. Proc. Natl. Acad. Sci. U.S.A. 101, 12491–12496. 10.1073/pnas.0403643101 15314214 PMC515087

[B23] Kumar S. Rosenberg J. M. Bouzida D. Swendsen R. H. Kollman P. A. (1992). The weighted histogram analysis method for free-energy calculations on biomolecules. I. The method. J. Comput. Chem. 13, 1011–1021. 10.1002/jcc.540130812

[B24] Lee K. H. Chen J. (2016). Multiscale enhanced sampling of intrinsically disordered protein conformations. J. Comput. Chem. 37, 550–557. 10.1002/jcc.23957 26052838

[B25] Lee M. S. Feig M. Salsbury F. R. Jr. Brooks C. L. III. (2003). New analytic approximation to the standard molecular volume definition and its application to generalized born calculations. J. Comput. Chem. 24, 1348–1356. 10.1002/jcc.10272 12827676

[B26] Lee M. S. Olson M. A. (2010). Protein folding simulations combining self-guided Langevin dynamics and temperature-based replica exchange. J. Chem. Theory Comput. 6, 2477–2487. 10.1021/ct100062b 26613500

[B27] Lee M. S. Olson M. A. (2011). Comparison of two adaptive temperature-based replica exchange methods applied to a sharp phase transition of protein unfolding-folding. J. Chem. Phys. 134, 244111–224417. 10.1063/1.3603964 21721616

[B28] Lee M. S. Salsbury F. R. Jr. Brooks C. L. III. (2002). Novel generalized born methods. J. Chem. Phys. 116, 10606–10614. 10.1063/1.1480013

[B29] Leung D. W. Borek D. Luthra P. Binning J. M. Anantpadma M. Liu G.. (2015). An intrinsically disordered peptide from Ebola virus VP35 controls viral RNA synthesis by modulating nucleoprotein-RNA interactions. Cell Rep. 11, 376–389. 10.1016/j.celrep.2015.03.034 25865894 PMC4599368

[B30] Mackerell A. D. Jr. Feig M. Brooks C. L. III. (2004). Extending the treatment of backbone energetics in protein force fields: limitations of gas-phase quantum mechanics in reproducing protein conformational distributions in molecular dynamics simulations. J. Comput. Chem. 25, 1400–1415. 10.1002/jcc.20065 15185334

[B31] Metropolis N. Rosenbluth A. Rosenbluth M. Teller A. Teller E. (1953). Equation of state calculations by fast computing machines. J. Chem. Phys. 21, 1087–1092. 10.1063/1.1699114

[B32] Miao Y. Feixas F. Eun C. McCammon J. A. (2015). Accelerated molecular dynamics simulations of protein folding. J. Comput. Chem. 36, 1536–1549. 10.1002/jcc.23964 26096263 PMC4487363

[B33] Mittal A. Lyle N. Harmon T. S. Pappu R. V. (2014). Hamiltonian switch Metropolis Monte Carlo simulations for improved conformational sampling of intrinsically disordered regions tethered to ordered domains of proteins. J. Chem. Theory Comput. 10, 3550–3562. 10.1021/ct5002297 25136274 PMC4132852

[B34] Olson M. A. Lee M. S. (2013). Structure refinement of protein model decoys requires accurate side-chain placement. Proteins 81, 469–478. 10.1002/prot.24204 23070940

[B35] Olson M. A. Lee M. S. (2014). Evaluation of unrestrained replica-exchange simulations using dynamic walkers in temperature space for protein structure refinement. PLoS ONE 9:e96638. 10.1371/journal.pone.0096638 24848767 PMC4029997

[B36] Olson M. A. Legler P. M. Goldman E. R. (2016). Comparison of replica exchange simulations of a kinetically trapped protein conformational state and its native form. J. Phys. Chem. B. 120, 2234–2240. 10.1021/acs.jpcb.6b00233 26886055

[B37] Perdigão N. Heinrich J. Stolte C. Sabir K. S. Buckley M. J. Tabor B.. (2015). Unexpected features of the dark proteome. Proc. Natl. Acad. Sci. U.S.A. 112, 15898–15903. 10.1073/pnas.1508380112 26578815 PMC4702990

[B38] Peter E. K. Shea J. E. Pivkin I. V. (2016). Coarse kMC-based replica exchange algorithms for the accelerated simulation of protein folding in explicit solvent. Phys. Chem. Chem. Phys. 18, 13052–13065. 10.1039/C5CP06867C 27111190

[B39] Phillips J. C. Braun R. Wang W. Gumbart J. Tajkhorshid E. Villa E.. (2005). Scalable molecular dynamics with NAMD. J. Comput. Chem. 26, 1781–1802. 10.1002/jcc.20289 16222654 PMC2486339

[B40] Piana S. Klepeis J. L. Shaw D. E. (2014). Assessing the accuracy of physical models used in protein-folding simulations: quantitative evidence from long molecular dynamics simulations. Curr. Opin. Struct. Biol. 24, 98–105. 10.1016/j.sbi.2013.12.006 24463371

[B41] Predescu C. Predescu M. Ciobanu C. V. (2004). The incomplete beta function law for parallel tempering sampling of classical canonical systems. Chem. Phys. 120, 4119–4128. 10.1063/1.1644093 15268578

[B42] Ryckaert J.-P. Ciccotti G. Berendsen H. J. C. (1977). Numerical integration of the Cartesian equations of motion of a system with constraints: molecular dynamics of n-alkanes. J. Comput. Phys. 23, 327–341. 10.1016/0021-9991(77)90098-5

[B43] Sanchez A. Geisbert T. W. Feldmann H. (2006). Filoviridae: Marburg and Ebola viruses, in Fields Virology, eds Knipe D. M. Howley P. M. Griffin R. A. Martin M. A. Roizman B. Straus S. E. (Philadelphia, PA: Lippincott Williams & Wilkins), 1409–1448.

[B44] Shoemaker B. A. Portman J. J. Wolynes P. G. (2000). Speeding molecular recognition by using the folding funnel: the fly-casting mechanism. Proc. Natl. Acad. Sci. U.S.A. 97, 8868–8873. 10.1073/pnas.160259697 10908673 PMC16787

[B45] Sugitaa Y. Okamoto Y. (1999). Replica-exchange molecular dynamics method for protein folding. Chem. Phys. Lett. 314, 141–151. 10.1016/S0009-2614(99)01123-9

[B46] Trebst S. Troyer M. Hansmann U. H. (2006). Optimized parallel tempering simulations of proteins. J. Chem. Phys. 124, 174903–174909. 10.1063/1.2186639 16689600

[B47] Wright P. E. Dyson H. J. (1999). Intrinsically unstructured proteins: re-assessing the protein structure-function paradigm. J. Mol. Biol. 293, 321–331. 10.1006/jmbi.1999.3110 10550212

[B48] Wright P. E. Dyson H. J. (2005). Intrinsically unstructured proteins and their functions. Nat. Rev. Mol. Cell Biol. 6, 197–208. 10.1038/nrm1589 15738986

[B49] Wu X. Brooks B. R. (2003). Self-guided Langevin dynamics simulation method. Chem. Phys. Lett. 381, 512–518. 10.1016/j.cplett.2003.10.013

[B50] Wu X. Brooks B. R. (2011). Toward canonical ensemble distribution from self-guided Langevin dynamics simulation. J. Chem. Phys. 134, 134108–134119. 10.1063/1.3574397 21476744 PMC3087419

[B51] Wu X. Brooks B. R. Vanden-Eijnden E. (2016). Self-guided Langevin dynamics via generalized Langevin equation. J. Comput. Chem. 37, 595–601. 10.1002/jcc.24015 26183423 PMC4715807

[B52] Wu X. Damjanovic A. Brooks B. R. (2012). Efficient and unbiased sampling of biomolecular systems in the canonical ensemble: a review of self-guided langevin dynamics. Adv. Chem. Phys. 150, 255–326. 10.1002/9781118197714.ch6 23913991 PMC3731171

[B53] Yeh I. C. Lee M. S. Olson M. A.. (2008). Calculation of protein heat capacity from replica-exchange molecular dynamics simulations with different implicit solvent models. J. Phys. Chem. B. 112, 15064–15073. 10.1021/jp802469g 18959439

[B54] Zhang W. Chen J. (2014). Replica exchange with guided annealing for accelerated sampling of disordered protein conformations. J. Comput. Chem. 35, 1682–1689. 10.1002/jcc.23675 24995857

